# Topical Application of A New Herbal Complex, NI-01, Ameliorates House Dust Mite-Induced Atopic Dermatitis in NC/Nga Mice

**DOI:** 10.3390/nu12051240

**Published:** 2020-04-27

**Authors:** Seong Eun Jin, Hyekyung Ha, Sae-Rom Yoo, Woo-Young Jeon, Nari Lee, Mee-Young Lee, Susanna Choi, Ji-Hye Jang, Eunsook Park, Sukkyoung Kim, Chang-Seob Seo

**Affiliations:** 1 Herbal Medicine Research Division, Korea Institute of Oriental Medicine, 1672 Yuseong-daero Yuseong-gu, Daejeon 34054, Korea; noellajin@kiom.re.kr (S.E.J.); hkha@kiom.re.kr (H.H.); k2aori@kiom.re.kr (S.-R.Y.); ssamggun85@kiom.re.kr (W.-Y.J.); nrlee0202@gmail.com (N.L.); cozy11@kiom.re.kr (M.-Y.L.); susannachoi@kiom.re.kr (S.C.); jihye1684@kiom.re.kr (J.-H.J.); eunsookpark@korea.kr (E.P.); 2 YM Bio, 397 Seokcheonro, Buchon, Gyeonggi-do 14449, Korea; skkim.ys@gmail.com

**Keywords:** atopic dermatitis, herbal complex, NC/Nga mice

## Abstract

Atopic dermatitis (AD) is a chronic inflammatory skin disease characterized by pruritus and cutaneous dry skin. Here, we investigated whether topical application of NI-01 composed of six herbal medicines has a therapeutic effect on AD in vivo. Twelve marker compounds of NI-01 were analyzed by high-performance liquid chromatography with a photodiode array detector for quality control. To induce AD, house dust mite extract was applied to the shaved dorsal skin and ear surfaces of NC/Nga mice twice a week for 6 weeks. NI-01 (1, 2, or 4 mg/mouse) was applied daily to the site for experiment periods. The coefficient of determination of each compound showed good linearity (≥ 0.9999). The recovery rate of the 12 marker components was 96.77%–105.17%; intra and interday precision and repeatability were ≤ 1.40%. Topical application of NI-01 reduced house dust mite induced AD symptoms. The increased expressions of interleukin-4 and intercellular adhesion molecule-1 caused by house dust mites were markedly suppressed in NI-01-treated mice. Corticosterone levels significantly decreased, whereas serotonin levels increased with NI-01 application. These results suggest that NI-01 alleviates AD symptoms by inhibiting infiltration of inflammatory cells, thereby decreasing AD-related stress. NI-01 could be beneficial for the treatment of AD-like skin diseases.

## 1. Introduction

Atopic dermatitis (AD), a chronic inflammatory skin disease, is characterized by severe pruritus, eczema, and cutaneous dry skin. The prevalence of AD continues to increase, and it affects approximately 15%–20% of children and 1%–3% of the adult population worldwide [1]. Several factors including genetic predisposition, environment, skin barrier, immune system dysregulation, and stress contribute to the pathogenesis of AD [2].

The central event in the maintenance and exacerbation of AD is pruritus, which triggers a vicious cycle of scratching and worsening of dermatitis [3]. Once the cycle of pruritus-scratch is initiated, inflammation of the skin is elevated by inducing keratinocytes to release a variety of pro-inflammatory cytokines. Moreover, this induces direction of lymphocytes, macrophages, and eosinophils into the inflammatory site [4].

In addition to inflammatory response and abnormalities of the immune system, psychological factors such as personality and stress play important roles in the exacerbation and chronicization of AD symptoms [3]. Patients with AD may exhibit immunological changes in response to stress, which may exacerbate inflammation and disease [3]. The fact that skin keratinocytes produce hormones, such as corticotropin-releasing hormone (CRH), adrenocorticotropic hormone (ACTH), and cortisol, and contain receptors for neurotransmitters, such as adrenaline, dopamine, and acetylcholine, indicates that there is a connection between the skin and psychoneuroimmunological pathways [5,6].

Typical therapies available to treat AD symptoms are predominantly corticosteroids and calcineurin inhibitors with T helper (Th) 2 cytokines inhibitory effects. However, these exhibit adverse effects such as skin atrophy, telangiectasia, striae, steroid rosacea, and tachyphylaxis with long-term use. In extreme cases, they carry a risk of serious side effects affecting adrenocortical dysfunction [7,8,9]. Therefore, natural product development to treat chronic inflammatory skin diseases such as AD is rising. In this study, we attempted to develop an effective and resistant natural product to AD, which is difficult to treat due to various etiologies and relapsing courses. 

Herbal medicine has long been used for treating various diseases, and more than 80% of the world’s population relies on it for healthcare [10]. A new herbal complex, NI-01, consists of six medicinal herbs, Arctii Fructus, Cinnamomi Ramulus, Lonicerae Flos, Moutan Radicis Cortex, Schisandrae Fructus, and Elsholtziae Herba, in equal ratio. Among these, Arctii Fructus (fruit of *Arctium lappa*), Moutan Radicis Cortex (root bark of *Paeonia suffruticosa*), Lonicerae Flos (flower bud of *Lonicera japonica*), and Cinnamomi Ramulus (twig of *Cinnamomum cassia*) have demonstrated anti-inflammatory effects [11,12,13,14]. In addition, Elsholtziae Herba (aerial parts of the genus *Elsholtzia*) and Schisandrae Fructus (fruit of *Schisandra chinensis*) have been reported to exhibit anti-mast cell-mediated allergic inflammation and immunostimulant activities, respectively [15,16]. It has been reported that *Cinnamomum cassia* reduces AD-like skin lesions by inhibiting Th2 cell response [17]. We hypothesized that NI-01, composed of these medicinal herbs, would also be effective in treating AD via anti-inflammatory, anti-allergic, and immunomodulatory effects.

In this study, for quality evaluation of NI-01, high performance liquid chromatography (HPLC) equipped with a photodiode array (PDA) detector was used to establish simultaneous analysis of marker components and validate the established analytical method. In addition, we assessed dermatitis severity, histological features, infiltration of inflammatory cells such as mast cells and CD4^+^ T cells, expression of interleukin (IL)-4 and intercellular adhesion molecule-1 (ICAM-1), and stress-related hormone and neurotransmitter levels for evaluation of the anti-AD effect of NI-01 in the house dust mite extract-induced AD model using NC/Nga mice.

## 2. Materials and Methods 

### 2.1. Plant Material

The six crude materials of NI-01 (Table 1) were purchased from Kyungdong Market (Seoul, Korea) and authenticated by Dr. Goya Choi, Herbal Medicine Resources Research Center, Korea Institute of Oriental Medicine (Daejeon, Korea). The voucher specimen (NI-01-1 through NI-01-6) was deposited at YM Bio (Bucheon, Korea).

### 2.2. Chemicals and Reagents

**Figure 1:**
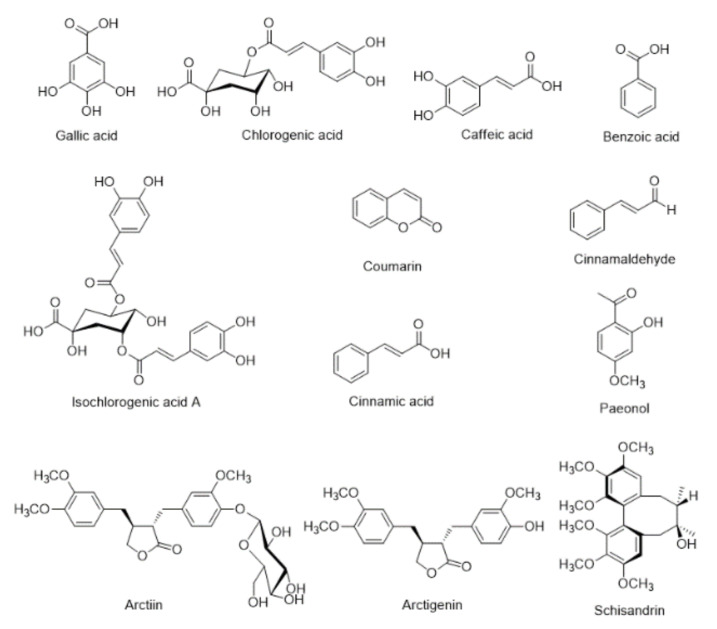
Chemical structures of 12 marker compounds in NI-01.

The 12 reference standards, gallic acid (PubChem CID: 370, purity 99.0%), chlorogenic acid (PubChem CID: 1794427, purity 99.6%), caffeic acid (PubChem CID: 689043, purity 99.0%), isochlorogenic acid A (PubChem CID: 6474310, purity 99.2%), benzoic acid (PubChem CID: 243, purity 99.9%), coumarin (PubChem CID: 323, purity 99.0%), arctiin (PubChem CID: 100528, purity 98.1%), cinnamic acid (PubChem CID: 444539, purity 99.5%), cinnamaldehyde (PubChem CID: 637511, purity 98.0%), paeonol (PubChem CID: 11092, purity 100.0%), arctigenin (PubChem CID: 64981, purity 99.4%), and schisandrin (PubChem CID: 23915, purity 100.0%) were purchased from the manufacturers of natural product standards: Merck KGaA (Darmstadt, Germany), Biopurify Phytochemicals (Chengdu, China), Acros Organics (Pittsburgh, PA, USA), Wako Chemicals (Osaka, Japan), and Shanghai Sunny Biotech (Shanghai, China), respectively (Figure 1). Organic solvents, methanol (PubChem CID: 887), acetonitrile (PubChem CID: 6342), and water (PubChem CID: 962), for HPLC analysis were of HPLC-grade and obtained from J. T. Baker (Phillipsburg, NJ, USA). Formic acid (ACS reagent, purity: ≥ 98.0%, PubChem CID: 284) was purchased from Merck KGaA (Darmstadt, Germany). Biostir-AD^®^, house dust mite (Dermatophagoides farinae) extract, was purchased from Biostir Inc. (Kobe, Japan). Prednisolone (Sigma-Aldrich, St. Louis, MO, USA) was used as a positive control. Enzyme-linked immunosolvent assay (ELISA) kits for measurement of immunoglobulin E (IgE), histamine, corticosterone, and serotonin were obtained from Abcam (Cambridge, UK & Cambridge, Massachusetts, USA), Oxford Biomedical Research Inc. (MI, USA), Enzo Life Sciences (Plymouth Meeting, PA), and Labor Diagnostika Nord GmbH & Co. KG (Nordhorn, Germany), respectively. A specific antibody against the CD4^+^ T cell was purchased from Abcam. Anti-IL-4, ICAM-1 and horseradish peroxidase-conjugated (HRP) secondary antibodies were purchased from Biorbyt Ltd. (Cowley Road, Cambridge, United Kingdom).

### 2.3. Preparation of NI-01 Water Extract

Six crude herbs, Cinnamomi Ramulus (2.0 kg), Lonicerae Flos (2.0 kg), Moutan Radicis Cortex (2.0 kg), Schisandrae Fructus (2.0 kg), Arctii Fructus (2.0 kg), and Elsholtziae Herba (2.0 kg), constituting NI-01 were mixed and extracted in water (120 L) at 100 °C for 4 h. The extracted solution was filtered and freeze-dried to give a powder sample. The amount of extracted NI-01 was 1.52 kg (12.7%).

### 2.4. Preparations of Sample and Standard Solutions

For simultaneous analysis of the 12 marker components (gallic acid, chlorogenic acid, caffeic acid, isochlorogenic acid A, benzoic acid, coumarin, arctiin, cinnamic acid, cinnamaldehyde, paeonol, arctigenin, and schisandrin) in NI-01, 40.0 mg of freeze-dried NI-01 extract was dissolved in 70% methanol (10 mL) and subsequently extracted using an ultrasonicator, Branson 8510 (Denbury, CT, USA) for 60 min at room temperature (25 °C). Thereafter, the extracted solution was filtered using a 0.2 μm membrane filter (Pall Life Sciences, Ann Arbor, MI, USA) prior to injection into the HPLC instrument. Standard solutions of the 12 reference standards were prepared at a concentration of 1.0 mg/mL using methanol and subsequently stored in a refrigerator maintained at 4 °C for further use.

### 2.5. Apparatus and Conditions

Simultaneous analysis of the 12 marker components for quality control of NI-01 was performed using a Shimadzu Prominence LC-20A system (Kyoto, Japan) equipped with a PDA detector and LabSolution software (Ver. 5.53, SP3, Kyoto, Japan) for the acquisition and processing of chromatographic data. The Gemini C18 column (4.6 × 250 mm, 5 μm; Phenomenex, Torrance, CA, USA) was used for separation of the 12 marker components and maintained at 40 °C. The mobile phase consists of distilled water (A) and acetonitrile (B), and both contained 0.1% (v/v) formic acid with a gradient condition as follows: 0–40 min, 5%–60% B; 40–50 min, 60%–100% B; 50–55 min, 100% B; 55–60 min, 100%–5% B; 60–70 min, 5% B. The flow rate of the mobile phase and injection volume of standard and sample solutions were maintained at 1.0 mL/min and 10 μL, respectively.

### 2.6. Validation of Analytical Procedures

Method validation (linearity, range, limit of detection (LOD), limit of quantification (LOQ), accuracy (recovery), and precision (repeatability, intraday, and interday)) of the optimal HPLC analytical method was carried out according to the International Conference Harmonisation guideline [18]. In other words, linearity of each compound was evaluated based on the coefficient of determination (r^2^) of the calibration curve. The tested linear range of each component was as follows: caffeic acid (0.16–10.00 μg/mL), isochlorogenic acid A, benzoic acid, coumarin, cinnamic acid, cinnamaldehyde, and schisandrin (0.31–20.00 μg/mL), gallic acid, chlorogenic acid, and arctigenin (0.78–50.00 μg/mL), and arctiin and paeonol (1.56–100.00 μg/mL). LOD and LOQ values were determined by the following equation. LOD = 3.3 × σ/S and LOQ = 10 × σ/S, where σ is the standard deviation of the y-intercept in each calibration curve and S is the slope of the calibration curve. A recovery test was conducted with a standard addition method to evaluate the accuracy and was calculated by the following equation; Recovery (%) = (founded amount – original amount)/spiked amount × 100. Intraday precision, interday precision (consecutive days; 1, 3, and 5 d, respectively), and repeatability were tested to verify the precision which was evaluated by the relative standard deviation (RSD). RSD was calculated by the following equation. RSD (%) = standard deviation (SD)/mean × 100.

### 2.7. Animals

Male NC/Nga mice (9 weeks old) were obtained from Central Laboratory Animal Inc. (Seoul, Korea) and acclimatized for 1 week prior to the start of the experiment. Animals were maintained in a temperature-controlled room at 23 ± 3 °C, relative humidity of ~40%–60%, and a 12 h light/dark cycle. Water and commercial rodent chow were provided ad libitum. This study was performed according to the NIH Guidelines for the Care and Use of Laboratory Animals [19], and the experiment was approved by the Institutional Animal Care and Use Committee of the Korea Institute of Oriental Medicine (Approval number: #18-007).

### 2.8. Induction of Atopic Dermatitis and Evaluation of Skin Severity

**Figure 2:**
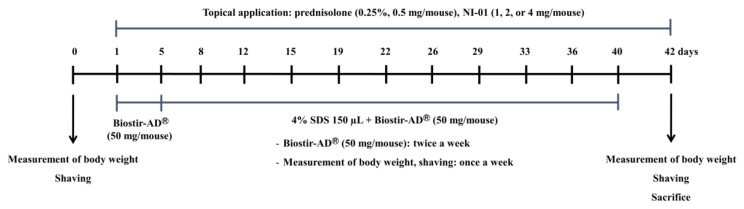
Summary of the experimental design.

Mice were divided into six groups (*n* = 8 animals/group) as follows: normal control group, Biostir-AD^®^ group, prednisolone group, and NI-01 group. Prednisolone and NI-01 were dissolved in 70% ethanol. The upper back of all mice was shaved one day prior to the experiment. The groups of normal control and Biostir-AD^®^ underwent application with 70% ethanol, and those in the positive control group underwent prednisolone application (0.25%, 0.5 mg/mouse) once daily for 6 weeks. NI-01 groups underwent NI-01 application (1, 2, or 4 mg/mouse) once daily for 6 weeks. To induce AD-like skin lesions, 150 μL of 4% (w/v) sodium dodecyl sulfate (SDS) was applied to shaved dorsal skin and both surfaces of each ear for barrier disruption. After 2 h, 50 mg of Biostir-AD^®^ was applied to all mice, except the normal control group, twice per week for 6 weeks (Figure 2). All mice were sacrificed with Entobar (pentobarbital sodium; Hanlim Pharm. Co., Ltd., Seoul, Republic of Korea). Blood was collected in BD Microtainer blood collection tubes containing EDTA-2K (Becton Dickinson Co., Franklin Lakes, NJ, USA) from abdominal veins. The dorsal skin and each ear were fixed in 4% paraformaldehyde for histopathological and immunohistochemical analysis. Dermatitis severity was assessed macroscopically every week in a blinded examination. Five signs, including pruritus/itching, erythema/hemorrhage, edema, excoriation/erosion, and scaling/dryness were scored as 0 (none), 1 (mild), 2 (moderate), and 3 (severe). The dermatitis score was defined as the sum of individual scores.

### 2.9. Measurement of Plasma IgE, Histamine, Corticosterone and Serotonin Levels

Plasma was separated (3000 rpm for 15 min at 4 °C) and stored at -80 °C for subsequent assays. Total IgE, histamine, corticosterone, and serotonin levels in plasma were measured using an ELISA kit following the manufacturer’s instructions.

### 2.10. Histological Analysis

Skin and ear tissue were fixed with 4% paraformaldehyde, embedded in paraffin, and sliced into 4-μm-thick sections by use of a Leica RM2255 Microtome (Leica Biosystems, Wetzlar, Germany). These sections were transferred to slides and stained with hematoxylin and eosin (H&E) or toluidine blue. Histopathological changes were assessed by use of Pannoramic^®^ DESK (3DHISTECH Ltd., Budapest, Hungary) and Pannoramic SlideScanner (Ver. 1.17.0, 3DHISTECH Ltd.) with a ×20 microscope objective. To determine mast cell infiltration into the upper dermis, mast cells of purple color were counted in three randomly selected areas of toluidine blue-stained sections.

### 2.11. Immunohistochemistry

Skin and ear slices were blocked in a blocking solution for 1 h and incubated with anti-CD4^+^ T cell, anti-IL-4, and anti-ICAM-1, which are primary antibodies, overnight at 4 °C. Thereafter, slices were linked with a horseradish peroxidase-conjugated secondary antibody for 1 h at room temperature (25 °C). Immunostained slides were confirmed by a Pannoramic^®^ DESK and visualized by a Pannoramic SlideScanner. For quantification of immunohistochemical results, image analysis was performed in three randomly selected areas using the Pannoramic Viewer (Ver. 1.15.4, 3DHISTECH Ltd., Budapest, Hungary) with a ×20 microscope objective.

### 2.12. Statistical Analysis

Data were expressed as the mean ± SEM. Statistical analyses were performed by one-way analysis of variance (ANOVA) followed by the Bonferroni post hoc test using SYSTAT (Ver. 10.0, Systat Software Inc., San Jose, CA, USA) and Student’s t-test. A value of *P* < 0.05 was considered statistically significant.

## 3. Results

### 3.1. Optimization of Chromatographic Conditions

For simultaneous analysis of the 12 marker components (gallic acid, chlorogenic acid, caffeic acid, isochlorogenic acid A, benzoic acid, coumarin, arctiin, cinnamic acid, cinnamaldehyde, paeonol, arctigenin, and schisandrin) in NI-01, various HPLC conditions, column types (e.g., Gemini C18, SunFire C18, Capcellpak UG120 C18, and OptimaPak C18 column), column temperatures (e.g., 30, 35, 40, and 45 °C), and mobile phase systems (e.g., formic acid and trifluoroacetic acid with acetonitrile) were tested. As a result, Gemini C18 column (250 mm × 4.6 mm, 5 μm), a column oven temperature of 40 °C, and mobile phase system of formic acid–acetonitrile were determined as optimal HPLC analysis conditions for simultaneous analysis of marker components.

### 3.2. Method Validations

As shown in Table 2, the calculated *r^2^* value at the tested concentration range of each component was ≥ 0.9999. These results show that the linearity of each calibration curve for each marker component is excellent. Also, LOD and LOQ values of all analytes were detected in concentrations of 0.05–0.41 μg/mL and 0.14–1.23 μg/mL, respectively (Table 2). Recovery of all analytes was 96.77%–105.17% and RSD values were less than 0.15% (Table 3). Furthermore, precisions (intraday, interday, and repeatability) were all less than 1.40% (Table 4). The above results suggest that the established HPLC analytical methods for simultaneous analysis of the 12 marker components in NI-01 are appropriate.

### 3.3. Simultaneous Determination of the 12 Marker Components in the Freeze-Dried NI-01 Sample

**Figure 3:**
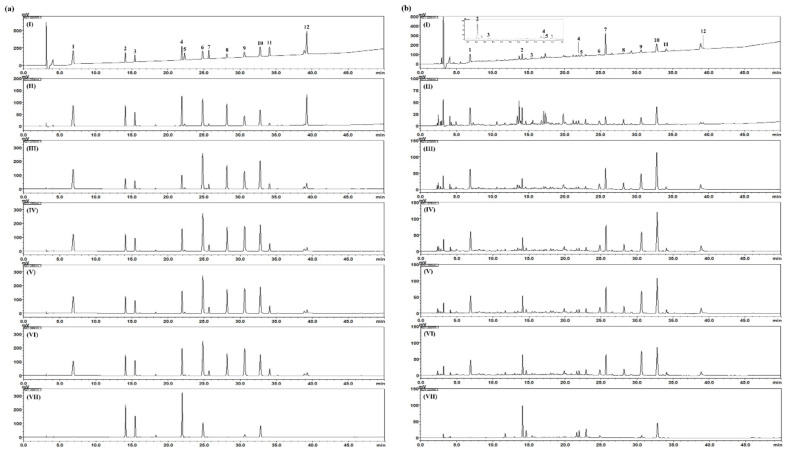
HPLC chromatograms of standard solutions (**a**) and NI-01 sample (**b**) at 225 nm (I), 254 nm (II), 270 nm (III), 275 nm (IV), 280 nm (V), 285 nM (VI), and 325 nm (VII). 1: gallic acid, 2: chlorogenic acid, 3: caffeic acid, 4: isochlorogenic acid A, 5: benzoic acid, 6: coumarin, 7: arctiin, 8: cinnamic acid, 9: cinnamaldehyde, 10: paeonol, 11: arctigenin, 12: schisandrin.

The established and validated HPLC analytical method was applied for simultaneous determination of the 12 marker components (gallic acid, chlorogenic acid, caffeic acid, isochlorogenic acid A, benzoic acid, coumarin, arctiin, cinnamic acid, cinnamaldehyde, paeonol, arctigenin, and schisandrin) for quality control of NI-01 sample. These 12 components were eluted at 6.87, 14.10, 15.44, 21.94, 22.33, 24.83, 25.69, 28.18, 30.62, 32.79, 34.09. and 39.26 min, respectively (Figure 3). The amounts of these components in freeze-dried NI-01 sample were detected 0.17 – 18.96 mg/g as shown in Table 5.

### 3.4. Effects of NI-01 on Dermatitis Score

**Figure 4:**
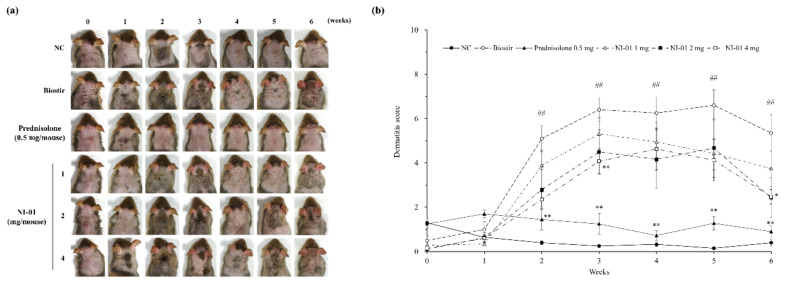
Effect of NI-01 on the severity of atopic dermatitis in house dust mite treated NC/Nga mice. Features of lesions (**a**) and dermatitis score (**b**) were assessed once a week for 6 weeks. Dermatitis scores were calculated as sums of individual scores, graded as 0 (none), 1 (mild), 2 (moderate), and 3 (severe) for the five signs: pruritus/itching, erythema/hemorrhage, edema, excoriation/erosion, and scaling/dryness. Prednisolone was used as a positive control. The data are expressed as the mean ± SEM (*n* = 8). ^##^*P* < 0.01 compared with the normal control group; ^*^P < 0.05 and ^**^P < 0.01 compared with the Biostir-AD^®^ group. NC; normal control, Biostir; Biostir-AD^®^ group.

In comparison to the normal control group, the Biostir-AD^®^-treated group revealed itching, erythema, and hemorrhage on the dorsal skin and ear that was followed by edema, excoriation, erosion, scaling, and dryness of the skin. As the study period progressed, clinical symptoms of dermatitis became more developed. In contrast, topical application of 0.25% (0.5 mg/mouse) prednisolone significantly reduced dermatitis symptoms compared with the Biostir-AD^®^ group (*P* < 0.01). The NI-01-treated groups showed improvements in AD signs and particularly the 4 mg/mouse of NI-01 significantly decreased the dermatitis score in comparison to the Biostir-AD^®^ group (*P* < 0.05, Figure 4).

### 3.5. Effect of NI-01 on Histopathological Features and Mast Cell Infiltration

**Figure 5:**
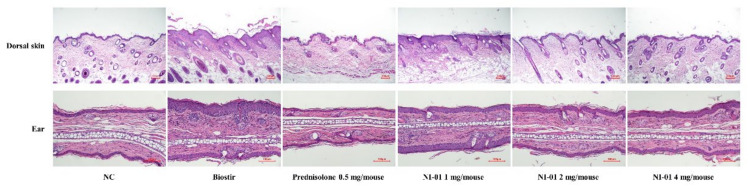
Effect of NI-01 on histological features of dorsal skin and ear lesions in house dust mite treated NC/Nga mice. Dorsal skin and ear tissue were fixed with 4% paraformaldehyde, embedded in paraffin and cut into thin sections. Histological features of lesions were determined with hematoxylin and eosin (×20). Prednisolone was used as a positive control. NC; normal control, Biostir; Biostir-AD^®^ group.

**Figure 6:**
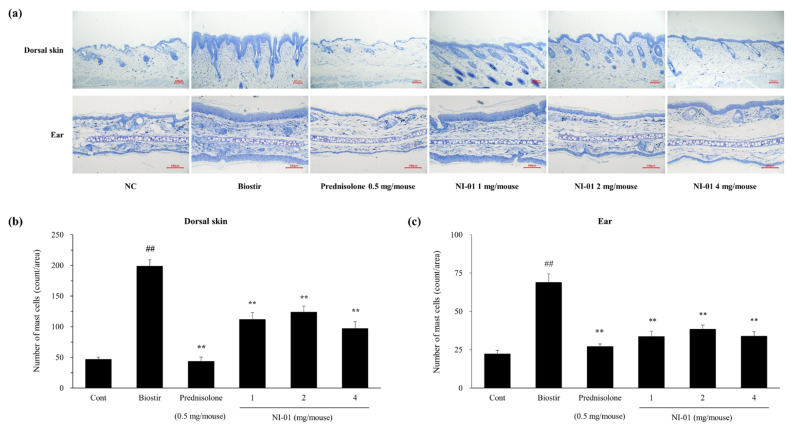
Effect of NI-01 on the mast cell infiltration in dorsal skin and ear lesions of house dust mite treated NC/Nga mice. Dorsal skin and ear tissues were fixed with 4% paraformaldehyde, embedded in paraffin and cut into thin sections. (**a**) Sections were stained with toluidine blue, and mast cells of purple color in dorsal skin (**b**) and ear (**c**) lesions were counted in three randomly selected areas under a microscope (×20). Prednisolone was used as a positive control. The data are expressed as the mean ± SEM (*n* = 8). ^##^*P* < 0.01 compared with the normal control group; ^**^P < 0.01 compared with the Biostir-AD^®^ group. NC; normal control, Biostir; Biostir-AD^®^ group.

In Figure 5, histological evaluation revealed that the dorsal skin and ear sections displayed hypertrophy, intracellular edema, and infiltration of inflammatory cells into the upper dermis in the Biostir-AD^®^-treated group compared with those in the normal control group. However, application of prednisolone or NI-01 (1, 2, and 4 mg/mouse) alleviated histological alterations compared to the Biostir-AD^®^ group (Figure 5). As shown in Figure 6, infiltration of mast cells in the dorsal skin and ear of the Biostir-AD^®^ group increased compared with the normal control group (*P* < 0.01). In contrast, the reduction of mast cell infiltration was observed in the 0.25% prednisolone and NI-01-treated groups (*P* < 0.01). Meanwhile, the levels of plasma IgE and histamine were higher in the Biostir-AD^®^ group than in the normal control group. However, application of NI-01 did not have an effect on IgE and histamine level reduction (Figure S1).

### 3.6. Effect of NI-01 on CD4^+^ T cell, IL-4, and ICAM-1 expression

**Figure 7:**
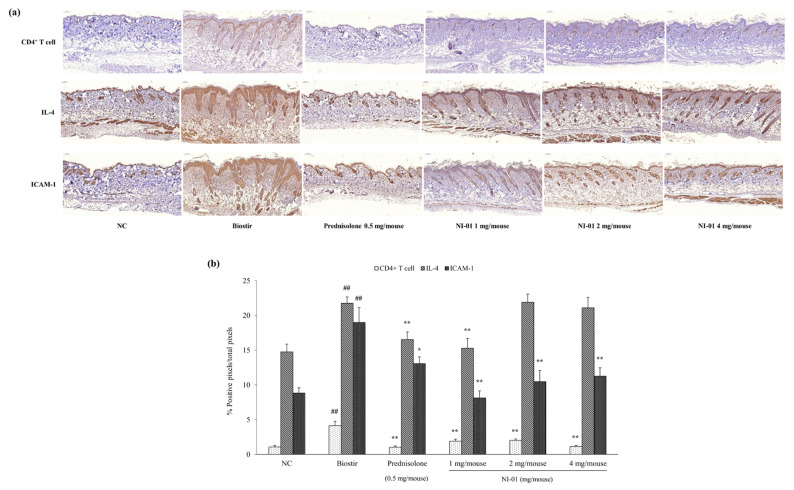
Immunohistochemical staining of dorsal skin lesions in house dust mite treated NC/Nga mice. Tissues were fixed with 4% paraformaldehyde, embedded in paraffin and cut into thin sections. (**a**) Sections were immunostained with anti-CD4^+^ T cell, IL-4, and ICAM-1 antibodies. Prednisolone was used as a positive control. (**b**) The intensity represents the mean ± SEM (*n* = 8). ^##^ *P* < 0.01 compared with the normal control group; ^*^ P < 0.05 and ^**^ *P* < 0.01 compared with the Biostir-AD^®^ group. NC; normal control, Biostir; Biostir-AD^®^ group.

To elucidate the anti-AD action mechanism of NI-01, immunohistochemical stainings of the CD4^+^ T cell, IL-4, and ICAM-1, in house dust mite treated NC/Nga mice were performed. As shown in Figure 7, the expression of CD4^+^ T cell, IL-4, and ICAM-1 significantly increased by Biostir-AD^®^ application (*P* < 0.01). In contrast, repeated application of prednisolone markedly reduced the expression of CD4^+^ T cell (*P* < 0.01), IL-4 (*P* < 0.01) and ICAM-1 (*P* < 0.05) was increased by Biostir-AD^®^. NI-01 (1, 2, and 4 mg/mouse) significantly reduced the CD4^+^ T cell and ICAM-1 expression compared to the Biostir-AD^®^ group (*P* < 0.01). Alternatively, IL-4 expression showed a significant decrease in the NI-01 1 mg/mouse group (*P* < 0.01), but did not change in NI-01 2 and 4 mg/mouse groups.

### 3.7. Effect of NI-01 on the Level of Corticosterone and Serotonin

**Figure 8:**
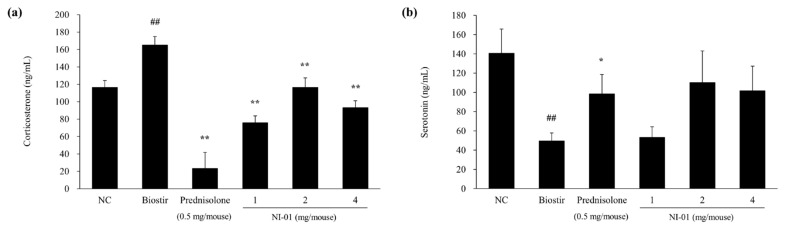
Effect of NI-01 on plasma levels of corticosterone (**a**) and serotonin (**b**) in house dust mite treated NC/Nga mice. Corticosterone and serotonin levels were measured by ELISA. Prednisolone was used as a positive control. The data are expressed as the mean ± SEM (*n* = 8). ^##^*P* < 0.01 compared with the normal control group; ^*^P < 0.05 and ^**^P < 0.01 compared with the Biostir-AD^®^ group. NC; normal control, Biostir; Biostir-AD^®^ group.

Application of Biostir-AD^®^ markedly increased the plasma level of corticosterone, while topical treatment with prednisolone and NI-01 (1, 2, and 4 mg/mouse) significantly decreased this level (P < 0.01) (Figure 8). The serotonin level in plasma was significantly decreased by Biostir-AD^®^ (P < 0.01), but evidently increased by prednisolone application (P < 0.05). Meanwhile, NI-01 (1, 2, and 4 mg/mouse) increased the plasma serotonin level compared to Biostir-AD^®^, but not significantly (Figure 8).

## 4. Discussion

The main findings of this study are that topical application of NI-01, consisting of six medicinal herbs, exhibited protective and therapeutic effects on AD, and suggest a method for standardization of NI-01. As the demands of herbal medicines are increasing globally, their quality standardization becomes more crucial. As herbal medicines contain relatively unrefined mixtures of phytochemicals, elucidation of compounds is important for quality control [20]. For standardization of NI-01, simultaneous analyses of the 12 marker compounds (gallic acid, benzoic acid, and paeonol, marker components of Moutan Radicis Cortex; chlorogenic acid, marker component of Lonicerae Flos and Arctii Fructus; caffeic acid, marker compound of Elsholtziae Herba; isochlorogenic acid A, arctiin, and arctigenin, marker components of Arctii Fructus; coumarin, cinnamic acid, and cinnamaldehyde, marker components of Cinnamomi Ramulus; schisandrin, marker compound of Schisandrae Fructus) contained in NI-01 were performed and validated based on linearity, range, LOD, LOQ, accuracy, and precision using a simple and universal HPLC instrument. These established HPLC methods could provide the basis for quality standardization of NI-01 or products containing NI-01.

To induce AD, we applied house dust mites to NC/Nga mice. NC/Nga mice have been extensively used as AD models [21] and pathological changes to their dermis have been reported as similar to those in AD patients [22]. To develop AD skin lesions under specific pathogen-free conditions, house dust mites, usually used to induce AD-like skin lesions in animal experiments, were repetitively applied to the dorsal skin and both surfaces of each ear in NC/Nga mice [23]. As a result, house dust mite induced AD mice showed severe physiological dysfunctions, including edema, excoriation, erosion, scaling, and dryness, as previously proposed [24]. By contrast, application of NI-01 markedly mitigated the severity of AD symptoms.

One of the key mechanisms of AD is imbalance of Th1/Th2 immune response through the secretion of Th2 cytokines such as IL-4 in skin lesions. The state of Th1/Th2 imbalance promotes degranulation of mast cells, and leads to the release of inflammatory mediators [25]. Mast cells play a crucial role in allergies and are particularly associated with itching. They regulate the immune system by releasing chemical mediators or by attracting other cellular entities involved in immune defense [26]. In addition, sensory nerve density in the epidermis and dermis has been reported to increase in AD-like skin lesions [26]. In this study, hyperplasia of the epidermis and dermis mast cell infiltration was markedly increased by house dust mites, but was significantly decreased by repetitive application of NI-01.

Several cytokines such as IL-4 have been reported to be involved in AD-associated pruritus [27]. We confirmed that massive infiltration of mast cells and CD4^+^ T cells as well as overexpression of IL-4 in skin lesions were induced by house dust mites, but were markedly reduced by topical application of NI-01. These results indicate that the decrease of epidermal hyperplasia and mast cell infiltration is related to the reduction of inflammatory mediators in the dermis.

Adhesion molecules are known to be involved in the infiltration of inflammatory cells at sites of inflammation. Among these adhesion molecules, ICAM-1, which is expressed in various skin diseases such as AD, contributes to the accumulation of mast cells in skin lesions [28]. In our results, the application of house dust mites caused the overexpression of ICAM-1 in skin lesions, whereas NI-01 treatment diminished them. These results indicate that NI-01 has anti-allergic and anti-inflammatory effects and can reduce Th2-related cytokines in AD.

Although several studies on AD have been conducted in relation to immunological viewpoints and skin barrier function, it is noteworthy that both stress and anxiety are associated with the secretion of neuropeptides, which release inflammatory mediators [21]. In effect, AD is associated with non-allergic diseases related to psychosocial factors or mental health [29,30], and high prevalence of anxiety and depression has been reported in AD patients [31]. Additionally, AD is exacerbated when patients undergo stressful events, and develops into a vicious cycle of pruritus, itching, and worsening of dermatitis [21]. We attempted to decipher the psychological factors closely related with AD symptoms.

Psychological stress is associated with changes in mast cells, neuropeptides, and cutaneous nerve fibers [26] as well as triggering complex immune pathways and stimulating the secretion of the CRH from the hypothalamus [6]. When CRH reaches the pituitary gland via circulation, it stimulates the secretion of ACTH to release cortisol [6]. Cortisol affects the immune system by inducing anti-inflammatory effects, regulating skin keratinocyte activity, disrupting skin barrier homeostasis, and increasing Th2 cell activity in chronic stress status [5,6]. Cortisol is the predominant adrenal stress hormone in humans, while corticosterone is the main corticosteroid hormone in rats and mice [32]. Thus, we confirmed that the level of corticosterone in plasma is increased by repetitive application of house dust mites, suggesting that AD affects stress. Serotonin, a neurotransmitter that regulates several functions such as behavior, endocrine processes, and sleep within the central nervous system [33], plays a key role in the pathophysiology of depression [34]. In addition, serotonin has been reported to modulate several major aspects of the immune system [33]. Here, application of house dust mites resulted in decreased serotonin concentration in mice plasma. These findings imply that AD patients are more likely to suffer from depression, which may worsen its prognosis. In addition, it is possible that stress has exacerbated AD by contributing to the induction of tissue inflammation via infiltration of inflammatory cells such as mast cells and CD4^+^ T cells. However, NI-01 treatment potentially altered the level of corticosterone and serotonin in plasma, suggesting that NI-01 may relieve AD symptoms via regulation of stress-related hormones and neurotransmitters.

The results of the current study show that topical application of NI-01 relieves AD symptoms of skin lesions, but does not affect plasma levels of histamine and IgE. In other words, repeated topical application of NI-01 is effective against skin lesions and has no effect on the body, suggesting no side effects such as systemic immunosuppression.

## 5. Conclusions

Topical application of NI-01 reduced clinical AD signs, epidermal hyperplasia and mast cell infiltration into the dermis. These effects are associated with a decrease of inflammatory factors in skin lesions, as well as alleviation of stress associated with AD symptoms. Our findings will be useful for the elucidation of the pathogenic mechanism and therapeutic targets in AD.

## Figures and Tables

**Table 1:** Composition of NI-01.

Herbal Medicine	Scientific Name	Family	Using Parts	Origin	Ratio (%)
Cinnamomi Ramulus	*Cinnamomum cassia* Presl	Lauraceae	Twigs	Vietnam	16.67
Lonicerae Flos	*Lonicera japonica* Thunberg	Caprifoliaceae	Flowers	China	16.67
Moutan Radicis Cortex	*Paeonia suffruticosa* Andrews	Paeoniaceae	Root barks	China	16.67
Schisandrae Fructus	*Schisandra chinensis* (Thrcz.) Baillon	Schisandraeceae	Fruits	Mungyeong, Korea	16.67
Arctii Fructus	*Arctium lappa* Linné	Compositae	Fruits	China	16.67
Elsholtziae Herba	*Elsholtzia ciliate* Hylander	Labiatae	Aerial parts	Yeongju, Korea	16.67
Total (%)					100

**Table 2:** Linear range, calibration curve, *r^2^*, limits of detections (LODs), and limits of quantification (LOQs) for marker compounds (*n* = 3).

Compound	Linear Range (μg/mL)	Slope	Intercept	*r* ^2^	LOD (μg/mL)	LOQ (μg/mL)
Gallic acid	0.78–50.00	36471.39	732.07	1.0000	0.23	0.69
Chlorogenic acid	0.78–50.00	29040.45	8.35	1.0000	0.18	0.53
Caffeic acid	0.16–10.00	62228.23	545.65	1.0000	0.05	0.14
Isochlorogenic acid A	0.31–20.00	38643.91	65.89	1.0000	0.08	0.24
Benzoic acid	0.31–20.00	41668.71	−55.39	1.0000	0.10	0.29
Coumarin	0.31–20.00	49231.58	214.54	1.0000	0.07	0.22
Arctiin	1.56–100.00	6823.74	890.85	1.0000	0.41	1.23
Cinnamic acid	0.31–20.00	82626.47	1747.26	1.0000	0.09	0.27
Cinnamaldehyde	0.31–20.00	149601.72	845.06	1.0000	0.06	0.18
Paeonol	1.56–100.00	58985.35	6937.15	1.0000	0.36	1.08
Arctigenin	0.78–50.00	9687.01	−378.67	1.0000	0.20	0.62
Schisandrin	0.31–20.00	20731.53	6609.43	0.9999	0.09	0.26

**Table 3:** Recovery test for the assay of 12 compounds in NI-01.

Compound	Original Conc. (μg/mL)	Spiked Conc. (μg/mL)	Found Conc. (μg/mL)	Recovery (%)	SD	RSD (%)
Gallic acid	16.07	3.00	19.04	99.08	0.78	0.78
		7.50	23.62	100.60	1.29	1.29
		15.00	31.08	100.07	0.72	0.72
Chlorogenic acid	19.73	4.00	23.65	98.15	0.14	0.15
		10.00	29.66	99.33	0.68	0.69
		20.00	40.76	105.17	0.81	0.77
Caffeic acid	0.66	1.00	1.66	99.30	0.82	0.82
		2.00	2.63	98.25	1.52	1.55
		4.00	4.57	97.64	0.92	0.94
Isochlorogenic acid A	3.98	1.00	4.98	99.69	1.77	1.78
		2.00	5.94	97.79	1.68	1.72
		4.00	7.95	99.20	0.70	0.71
Benzoic acid	2.83	1.00	3.83	99.38	1.19	1.19
		2.00	4.86	101.13	0.68	0.67
		4.00	6.97	103.38	0.88	0.85
Coumarin	3.70	1.00	4.71	101.36	0.59	0.58
		2.00	5.66	98.18	1.44	1.46
		4.00	7.67	99.41	0.59	0.59
Arctiin	75.10	15.00	89.90	98.63	1.01	1.02
		37.50	112.21	98.95	1.71	1.73
		75.00	148.97	98.49	1.15	1.17
Cinnamic acid	2.20	1.00	3.23	102.45	2.03	1.99
		2.00	4.21	100.29	1.46	1.45
		4.00	6.30	102.30	0.71	0.70
Cinnamaldehyde	5.11	1.00	6.11	100.15	0.76	0.76
		2.00	7.09	99.24	1.27	1.28
		4.00	9.07	99.09	0.59	0.60
Paeonol	21.76	4.00	25.78	100.56	1.25	1.24
		10.00	31.65	98.85	1.00	1.02
		20.00	41.12	96.77	0.26	0.27
Arctigenin	10.11	2.00	12.11	100.04	0.79	0.79
		5.00	15.10	99.85	1.39	1.39
		10.00	20.10	99.92	0.62	0.62
Schisandrin	1.04	1.00	2.04	100.52	1.44	1.44
		2.00	3.00	98.07	0.19	0.20
		4.00	5.07	100.87	0.98	0.97

**Table 4:** Precision assay for 12 marker compounds in NI-01.

Compound	Conc. (μg/mL)	Intraday (*n* = 5)			Interday (*n* = 5)			Repeatability (*n* = 6)	
		Observed Conc. (μg/mL)	Precision (%)	Accuracy (%)	Observed Conc. (μg/mL)	Precision (%)	Accuracy (%)	RSD (%) of Retention Time	RSD (%) of Peak Area
Gallic acid	12.50	12.71	0.07	0.52	101.65	12.60	0.16	0.07	0.46
	25.00	25.15	0.21	0.85	100.60	25.19	0.30		
	50.00	50.24	0.45	0.89	100.47	50.30	0.62		
Chlorogenic acid	12.50	12.69	0.07	0.52	101.48	12.57	0.16	0.04	0.31
	25.00	25.09	0.21	0.83	100.38	25.11	0.30		
	50.00	50.28	0.46	0.92	100.57	50.23	0.70		
Caffeic acid	2.50	2.54	0.02	0.70	101.73	2.52	0.03	0.04	0.46
	5.00	5.03	0.04	0.78	100.69	5.04	0.06		
	10.00	10.06	0.11	1.09	100.59	10.07	0.13		
Isochlorogenic acid A	5.00	5.07	0.02	0.42	101.47	5.02	0.07	0.03	0.42
	10.00	10.05	0.07	0.65	100.50	10.03	0.10		
	20.00	20.11	0.20	0.99	100.53	20.05	0.24		
Benzoic acid	5.00	5.09	0.06	1.11	101.77	5.03	0.07	0.03	0.10
	10.00	10.06	0.12	1.19	100.64	10.04	0.12		
	20.00	20.11	0.19	0.94	100.54	20.02	0.20		
Coumarin	5.00	5.10	0.04	0.73	101.93	5.05	0.06	0.02	0.40
	10.00	10.04	0.09	0.85	100.40	10.07	0.12		
	20.00	20.12	0.21	1.05	100.62	20.09	0.22		
Arctiin	25.00	25.49	0.16	0.61	101.96	25.29	0.33	0.03	0.44
	50.00	50.43	0.44	0.86	100.85	50.54	0.65		
	100.00	100.56	1.10	1.10	100.56	100.69	1.36		
Cinnamic acid	5.00	5.08	0.03	0.61	101.70	5.04	0.06	0.03	0.38
	10.00	10.05	0.09	0.85	100.52	10.08	0.12		
	20.00	20.10	0.19	0.97	100.52	20.08	0.18		
Cinnamaldehyde	5.00	5.08	0.03	0.62	101.53	5.04	0.06	0.03	0.37
	10.00	10.01	0.08	0.80	100.09	10.06	0.11		
	20.00	20.11	0.17	0.86	100.57	20.09	0.22		
Paeonol	25.00	25.37	0.16	0.63	101.49	25.19	0.26	0.03	0.40
	50.00	50.27	0.43	0.85	100.55	50.41	0.60		
	100.00	100.52	0.93	0.93	100.52	100.36	1.10		
Arctigenin	12.50	12.66	0.08	0.61	101.26	12.55	0.16	0.03	0.41
	25.00	25.26	0.22	0.85	101.04	25.22	0.33		
	50.00	50.26	0.53	1.05	100.53	50.29	0.62		
Schisandrin	5.00	5.10	0.03	0.68	101.98	5.08	0.06	0.02	0.36
	10.00	10.07	0.10	1.01	100.72	10.12	0.13		
	20.00	20.04	0.14	0.72	100.21	20.06	0.18		

**Table 5:** The amount of 12 marker compounds in the freeze-dried NI-01 sample (n = 3).

Compound	Batch No. Concentrations (mg/g) ± SD (× 10^−2^)		
	1	2	3
Gallic acid	3.90 ± 1.03	3.89 ± 0.84	3.89 ± 0.11
Chlorogenic acid	4.92 ± 0.26	4.92 ± 1.09	4.93 ± 0.34
Caffeic acid	0.17 ± 0.09	0.17 ± 0.16	0.17 ± 0.10
Isochlorogenic acid A	1.00 ± 0.29	1.00 ± 0.12	1.00 ± 0.03
Benzoic acid	0.88 ± 0.76	0.88 ± 0.15	0.87 ± 0.89
Coumarin	0.92 ± 0.65	0.92 ± 0.09	0.92 ± 0.13
Arctiin	18.95 ± 0.94	18.93 ± 0.45	18.96 ± 1.13
Cinnamic acid	0.56 ± 0.15	0.56 ± 0.14	0.56 ± 0.13
Cinnamaldehyde	1.29 ± 0.28	1.29 ± 0.65	1.29 ± 0.51
Paeonol	5.46 ± 0.66	5.47 ± 0.75	5.46 ± 1.13
Arctigenin	2.48 ± 6.41	2.52 ± 6.15	2.49 ± 4.95
Schisandrin	0.25 ± 0.23	0.25 ± 0.40	0.25 ± 0.51

## Supplementary Materials

The following are available online at https://www.mdpi.com/2072-6643/12/5/1240/s1, Figure S1: Effect of NI-01 on plasma levels of IgE (**a**) and histamine (**b**) in house dust mite treated NC/Nga mice. IgE and histamine levels were measured by ELISA. Prednisolone was used as a positive control. The data are expressed as the mean ± SEM (*n* = 8). ^##^*P* < 0.01 compared with the normal control group. NC; normal control, Biostir; Biostir-AD^®^ group.
