# Supplementary material for: Topical Application of A New Herbal Complex, NI-01, Ameliorates House Dust Mite-Induced Atopic Dermatitis in NC/Nga Mice
Source: Nutrients. 2020 Apr 27;12(5):1240. doi: 10.3390/nu12051240 (PMC7284439; doi:10.3390/nu12051240)
Supplement: Supplementary file 1 [file nutrients-12-01240-s001.zip › nutrients-767693-supplementary.docx]

| 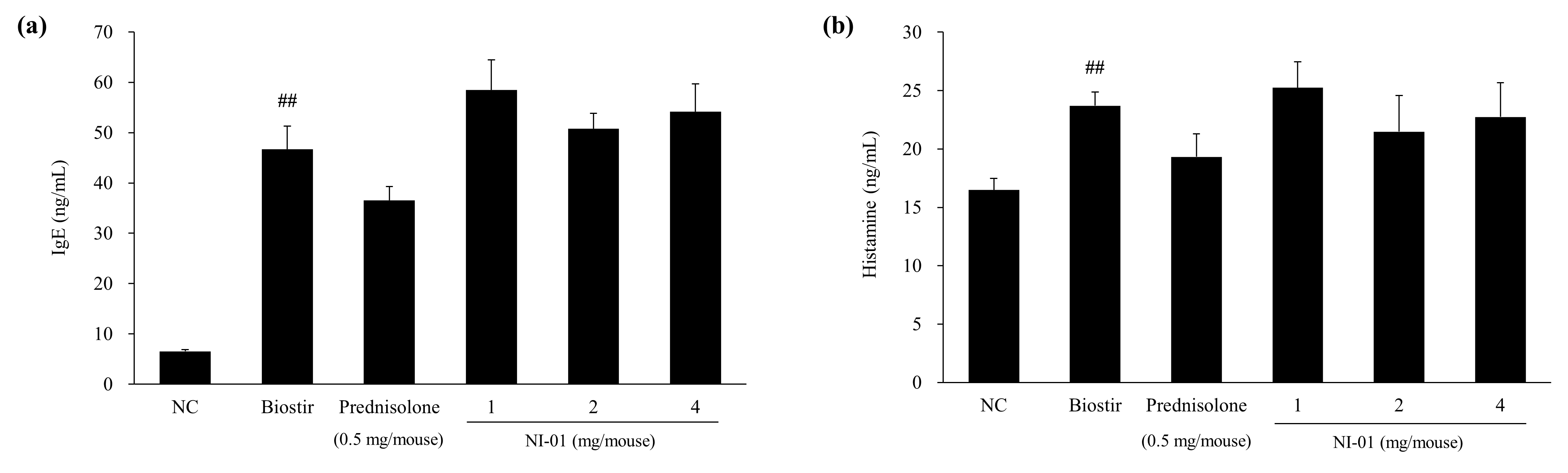 |
| --- |

**Figure S1.** Effect of NI-01 on plasma levels of IgE **(a)** and histamine **(b)** in house dust mite treated NC/Nga mice. IgE and histamine levels were measured by ELISA. Prednisolone was used as a positive control. The data are expressed as the mean ± SEM (n = 8). ^##^*P* < 0.01 compared with the normal control group. NC; normal control, Biostir; Biostir-AD^®^ group.
